# Green and Mild Fabrication of Magnetic Poly(trithiocyanuric acid) Polymers for Rapid and Selective Separation of Mercury(II) Ions in Aqueous Samples

**DOI:** 10.3390/polym16213067

**Published:** 2024-10-31

**Authors:** Qianqian Li, Boxian Ruan, Yue Yu, Linshu Ye, Aoxiong Dai, Sasha You, Bingshan Zhao, Limin Ren

**Affiliations:** Hubei Key Laboratory of Processing and Application of Catalytic Materials, Department of Chemistry, Huanggang Normal University, Huangzhou 438000, China; liqianqian@hgnu.edu.cn (Q.L.);

**Keywords:** magnetic sorbent, porous organic polymer, green and mild reaction, adsorption and separation of mercury(II) ions, magnetic solid-phase extraction, trace elemental analysis

## Abstract

The removal and detection of highly toxic mercury(II) ions (Hg^2+^) in water used daily is essential for human health and monitoring environmental pollution. Efficient porous organic polymers (POPs) can provide a strong adsorption capacity toward heavy metal ions, although the complex synthetic process and inconvenient phase separation steps limit their application. Hence, a combination of POPs and magnetic nanomaterials was proposed and a new magnetic porous organic polymer adsorbent was fabricated by a green and mild redox reaction in the aqueous phase with trithiocyanuric acid (TA) and its sodium salts acting as reductive monomers and iodine acting as an oxidant. In the preparation steps, no additional harmful organic solvent is required and the byproducts of sodium iodine are generally considered to be non-toxic. The resulting magnetic poly(trithiocyanuric acid) polymers (MPTAPs) are highly porous, have large surface areas, are rich in sulfhydryl groups and show easy magnetic separation ability. The experimental results show that MPTAPs exhibit good adsorption affinity toward Hg^2+^ with high selectivity, rapid adsorption kinetics (10 min), a large adsorption capacity (211 mg g^−1^) and wide adsorption applicability under various pH environments (pH 2~8). Additionally, MPTAPs can be reused for up to 10 cycles, and the magnetic separation step of MPTAPs is fast and convenient, reducing energy consumption compared to centrifugation and filtration steps required for non-magnetic adsorbents. These results demonstrate the promising capability of MPTAPs as superior adsorbents for effective adsorption and separation of Hg^2+^. Based on this, the prepared MPTAPs were adopted as magnetic solid-phase extraction (MSPE) materials for isolation of trace Hg^2+^ from aqueous samples. Under optimized conditions, the extraction and quantification of trace Hg^2+^ in water samples were accomplished using inductively coupled plasma mass spectrometry (ICP-MS) detection after MSPE procedures. The proposed MPTAPs-based MSPE-ICP-MS method is efficient, rapid, sensitive and selective for the determination of trace Hg^2+^, and was successfully employed for the accurate analysis of trace Hg^2+^ in tap water, wastewater, lake water and river water samples.

## 1. Introduction

The heavy metal element mercury (Hg) was previously used extensively in metallurgy, electrolyzation, medicine, electronics, etc. [1]. However, environmental pollution, especially water pollution, caused by Hg-related industry is still a current issue. Hg-polluted water not only causes harm to aquatic animals and plants, but also impacts human health through impacting the whole biological chain. The World Health Organization recommends that the maximum allowable concentration of Hg in potable water should be lowered to 1 μg L^−1^ [2]. The Environmental Protection Administration of China also regulates guidelines for Hg in different surface waters (recommending below 0.05 and 0.1 μg L^−1^ for potable water sources and surface waters used daily, respectively) and drinking water (recommending below 1 μg L^−1^) [3,4]. Hence, the removal of excessive Hg and the precise and sensitive measurement of trace Hg in water samples are crucial for ensuring water quality.

Inductively coupled plasma mass spectrometry (ICP-MS) is a prevalent analytical tool renowned for its exceptional sensitivity in the detection of trace elements, and it has been frequently used in water quality surveillance [5]. However, the direct analysis of target elements in real samples by ICP-MS is often faced with difficulties such as matrix interferences and low levels of analytes [6]. To address this problem, efficient solid sorbents and solid-phase extraction (SPE) procedures are often employed before using instruments for the analytical step, and can separate complex matrices and enrich target analytes.

Nowadays, various solid sorbents such as carbon-based materials [7], ion-imprinted polymers [8], porous organic polymers (POPs) [9] and covalent organic frameworks (COFs) [10] have been utilized in the extraction of trace target analytes. It should be highlighted that POPs are a superior class of solid sorbents that possess polymer networks, which are constructed by organic monomers containing light elements. These polymer networks usually have extensive surface areas, show high porosity, are low-weight and show good stability. To date, various POPs have been fabricated and utilized for the adsorption of different metal ions [11,12,13,14,15,16,17]. However, phase separation is often difficult because of the low weight and density of POPs, which necessitates time-consuming steps like filtration or centrifugation. Introducing magnetic nanomaterials into the polymers is one way to overcome the separation difficulty, as the magnetic nanomaterials are easily attracted and isolated from the liquid phase under an additional magnetic field. However, POPs are typically fabricated under stringent conditions, such as several days of high-temperature reactions involving the employment of expensive catalysts and a significant amount of organic solvents. This complexity poses challenges in synthesizing magnetic POPs (MPOPs) under milder conditions, thereby restricting the practical application of MPOPs. Fortunately, some endeavors have been undertaken to address this limitation, including the proposal of mild synthesis methods for POPs [18,19,20,21,22,23]. Through employing gentle diazo coupling reactions between different diazonium salts and phenols in aqueous solution, Huang et al. successfully prepared -SH-modified MPOPs for the effective removal of heavy metal ions [21] and MPOPs rich in phenolic -OH groups to achieve fast scavenging of organic dyes [22]. By using a cheap and non-toxic metal chelator, trimercaptos-triazine-trisodium salt, Xiao et al. [23] synthesized a nanoporous polymer sorbent through an oxidation–reduction reaction in aqueous solution. The synthetic procedure is straightforward and eliminates the use of environmentally unfriendly organic solvents, making this method exceptionally appealing for the preparation of MPOPs. These innovative approaches underscore the versatility and environmental compatibility of mild synthesis methods in tailoring magnetic polymeric materials for diverse applications.

Inspired by these works above, in this study, we utilize an oxidation–reduction reaction to construct MPOPs in an aqueous environment by using trithiocyanuric acid (TA) and its salt as organic precursors in the presence of magnetic particles. All the ingredients used in the synthesis process were inexpensive and had low toxicity. No additional harmful organic solvents were required and no harmful byproducts were produced; thus, the synthetic process is cost-effective, green and mild. The resulting magnetic poly(trithiocyanuric acid) polymers (MPTAPs) show easy magnetic separation ability, are highly porous and have large specific surface areas. Adsorption experiment results show that MPTAPs exhibit high selectivity, a rapid adsorption dynamic and great adsorption ability toward mercury(II) ions (Hg^2+^). Thus, MPTAPs were adopted as magnetic solid-phase extraction (MSPE) adsorbents for the effective extraction of trace Hg^2+^ from real water samples, followed by ICP-MS determination.

## 2. Materials and Methods

### 2.1. Chemicals and Apparatus

FeCl_3_·6H_2_O (99%), FeCl_2_·4H_2_O (98%), aqueous ammonia (28%), sodium hydroxide (95%), concentrated HCl solution (37%) and ethanol (absolute) were purchased from SINOPHARM (Shanghai, China). TA (95%), tetraethoxysilane (TEOS, 98%), 1,3,5-triazine-2,4,6-trithiol trisodium salt (TTS, 98%), potassium iodide (99%), iodine (99.8%), 5,5-dithiobis-2-nitrobenzoic acid (DTNB) and thiourea (99%) were purchased from Aladdin Reagent (Shanghai, China). The standard solutions of various ions (Hg^2+^, Cu^2+^, Zn^2+^, Co^2+^, Ni^2+^, Cd^2+^, Pb^2+^, Cr^3+^ etc.) and the certified reference material of GSB07-3173-2014 (202061) standard water sample were purchased from Tan-Mo Company (Beijing, China).

The deionized water was acquired from the UPT-I-10T pure water instrument (You-Pu, Chengdu, China). The PHS-3E pH meter was purchased from Lei-Ci company (Shanghai, China) and utilized for measuring pH values. The LC-P1 vortex mixer and HY-2A shaker were purchased from Li-Chen company (Shanghai, China) and applied as a mixing solution. The KQ-5200 DE ultrasonic instrument was purchased from Kun-Shan company (Kunshan, China) and employed for dispersing mixed solution. The Nd_2_Fe_14_B magnets (50 × 30 × 10 mm) were acquired from Heng-Xin magnet company (Dongguan, China) and employed for magnetic separation. The MCE membrane filters (50 mm × 0.45 µm) were purchased from Jing-Teng company (Tianjing, China) and used for filtration of aqueous solution. ICP-MS 2030 (Shimadzu, Japan) was employed for the determination of target elements and the instrumental working parameters are shown in Table S1 in Supplementary Materials. The infra-red spectrograms and magnetism of the prepared materials were obtained from a Nicolet-iS10 Fourier transform infrared spectrometer (FT-IR, Thermo, Waltham, MA, USA) and a PPMS-9T vibrating sample magnetometer (VSM, Quantum Design, San Diego, CA, USA), respectively. The InVia Raman spectrometer (Renishaw, London, UK) was applied for the characterization of special groups. The TU-1950 ultra-violet and visible spectrophotometer (UV-vis, Shanghai, China) was employed for the quantification of sulfhydryl groups in MPTAPs by the Ellman method [24]. The SDTQ600 thermal gravimetric analyzer (TG, TA, Santa Fe Springs, CA, USA) was applied for the measurement of the thermal stability. The XRD-6000 X-ray diffractometer (Shimadzu, Kyoto, Japan) was employed for the characterization of the crystal structure. The surface morphology and pore structure of MPTAPs were characterized by the FEI Tecnai F20 transmission electron microscope (TEM, FEI, San Jose, CA, USA) and the ASAP 2460 automated specific surface area and pore size analyzer (Micromeritic, Norcross, GA, USA), respectively.

### 2.2. Preparation of MPTAPs

**Figure 1:**
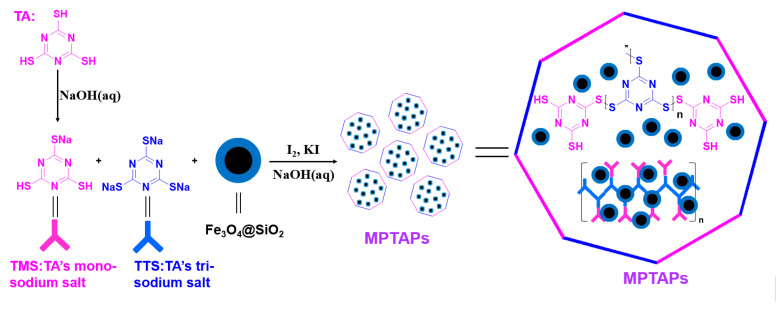
Synthetic process of MPTAPs. (The red and blue lines severally represent the TMS and TTS, the coloured framework composed of alternating red and blue lines represents the disulfide polymer network structure constructed by TMS and TTS.)

Initially, Fe_3_O_4_@SiO_2_ magnetic nanoparticles (MNPs) were synthesized (refer to the reported research [25]), and the detailed process is listed in Appendix SA in Supplementary Materials. Subsequently, Fe_3_O_4_@SiO_2_ MNPs were decorated with organic disulfide polymer in aqueous phase. The detailed fabrication process is as follows. A total of 177 mg (1 mmol) TA was added into 60 mL ultrapure water, and the solution was stirred evenly and added into a three-necked round-bottomed flask followed by mechanical agitation. Then, 10 mL of 0.1 mol L^−1^ NaOH solution was slowly added under mechanical stirring, and the mixed solution was continuously stirred for 2 h in a water bath (room temperature). After that, the solution of trimercaptos-triazine-monosodium salt (TA’s monosodium salt, TMS) was formed, which was denoted as solution A. At the same time, 243 mg (1 mmol) TTS (TA’s trisodium salt) was dissolved in 50 mL ultrapure water and 200 mg Fe_3_O_4_@SiO_2_ MNPs were added and dispersed ultrasonically for 10 min. The obtained homogeneous solution was denoted as solution B and added to solution A with rapid stirring. Finally, 10 mL concentrated aqueous solution of potassium iodide containing 508 mg iodine was slowly added into the mixed solution of A and B under ice-water bath at 5~10 °C. The added iodine acted as an oxidizing agent to oxidize the active sodium sulfide salt in TMS and TTS to connective disulfide compounds and the reductive byproducts of sodium iodide were produced in the solution. The low-temperature environment prevents iodine from evaporating and reacting too quickly. After the addition of iodine was finished, no additional ice was added in ice-water bath and the mixture was kept under mechanical stirring for 12 h at room temperature without a mechanical heating source, and then the mixture solution gradually warmed to room temperature. This operation ensures that the temperature slowly rises to room temperature; thus, the volatilization of iodine is significantly reduced and the redox reaction is gradually performed, and the generated connective disulfide polymers gradually wrap around Fe_3_O_4_@SiO_2_ particles. After the reaction, the gray magnetic solids were obtained, and these were denoted as MPTAPs. The obtained MPTAPs were washed with HCl (0.2 mol L^−1^) and water several times, then dried at 45 °C within a vacuum oven. Figure 1 illustrates the synthetic process of MPTAPs. According to the reaction mechanism, the mass of reactant and obtained MPTAPs, the yield of MPTAPs was calculated to be 93%, which indicates a complete reaction between the organic monomers. Compared with some POPs synthesized by a tedious synthetic procedure (24~72 h) with severe conditions and expensive monomers [13,15,26,27], MPTAPs can be fabricated rapidly under milder conditions with low-cost non-toxic reagents and no additional harmful organic solvents are required. Furthermore, the byproduct of sodium iodine is generally considered to be non-toxic. Thus, the synthetic process of MPTAPs is thought to be low-cost, green and mild.

### 2.3. Adsorption Experiments

A certain concentration of metal ion solution was prepared after dilution of corresponding standard solution, and then the solution pH was adjusted by diluted HCl and NaOH. A total of 50 mL of the prepared solution was poured into a beaker, followed by the addition of a certain mass of the MPTAP sorbent, and then the mixture was agitated for a duration ranging from 1 to 20 min. After the adsorption step, a magnet was placed under the beaker and the MPTAP sorbent was quickly attracted to the bottom of the beaker, and then the supernatant liquid was collected. The content of metal ions in the solution, both prior to and after the adsorption process, was measured using ICP-MS.

### 2.4. Sample Preparation

Industrial acidic wastewater samples were collected from Huanggang TCL Environmental Technology Co., Ltd. (Huanggang, China). Tap water and laboratory wastewater samples were obtained from the chemistry laboratory in Huanggang Normal University, located in Huanggang, China. River water and lake water samples were obtained from the Yangtze River (Huanggang section ferry, China) and the Hongzhu Lake (Huanggang, China), respectively. All the real water samples were pre-filtered to eliminate the solid particles.

### 2.5. MSPE-ICP-MS Procedure

A 100 mL aliquot of the sample solution was introduced into a beaker, followed by the adjustment of the solution pH to 6, the addition of 10 mg MPTAPs, and the agitation of mixed solution for 10 min in sequence. Subsequently, MPTAP materials were isolated by introducing a magnet under the beaker, and the supernatant was discarded. After the adsorption procedure, 0.4 mL of 0.5 mol L^−1^ HCl solution with 0.4% thiourea was introduced and continuously vortexed for 3 min to elute the target ions from the MPTAPs. Lastly, the eluate liquid was isolated by a magnet and then transferred to the ICP-MS for determination.

## 3. Results

### 3.1. Characterization

**Figure 2:**
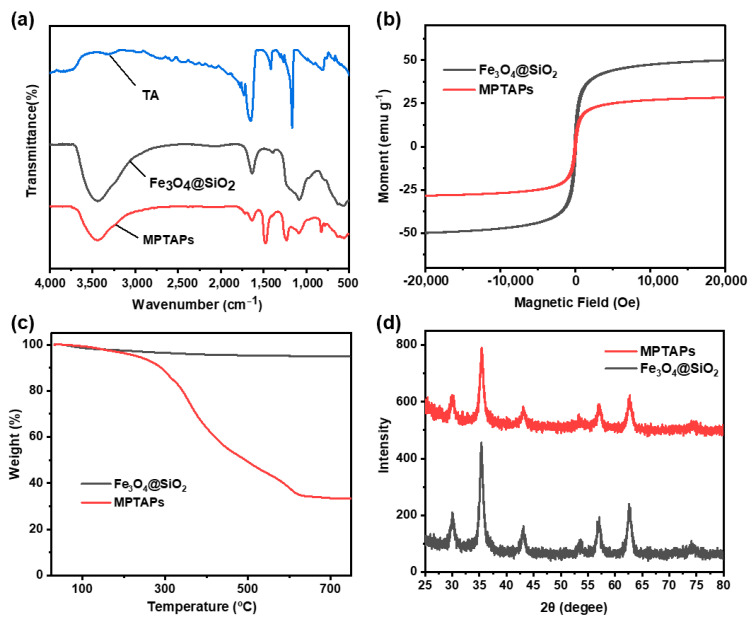
FT-IR (**a**), VSM (**b**), TG (**c**) and XRD (**d**) analysis for Fe_3_O_4_@SiO_2_ and MPTAPs. (The black and red lines represent the characterization results of Fe_3_O_4_@SiO_2_ and MPTAPs, respectively.)

Firstly, the self-prepared Fe_3_O_4_@SiO_2_ and MPTAPs were analyzed by FT-IR, with the spectral data recorded between 400 and 4000 cm^−1^ (Figure 2a). In the IR spectrum of Fe_3_O_4_@SiO_2_, the absorption peaks observed around 1080 cm^−1^ and 570 cm^−1^ correspond to the stretching vibrations of Si-O and Fe-O bonds, respectively, confirming the successful synthesis of Fe_3_O_4_@SiO_2_. For MPTAPs, the peaks located at approximately 1450, 1251 and 829 cm^−1^ are indicative of the characteristic absorption peaks for triazine groups [23]. It is difficult to observe the characteristic stretching vibration peak of sulfhydryl groups (-SH) in the figure (usually around 2600~2500 cm^−1^), which may be related to the weak strength of their absorption peaks. To further confirm the existence of -SH on MPTAPs, the Ellman method [24] with a chromogenic reagent (DTNB) was employed to quantify the sulfhydryl groups in the sorbent. The DTNB solution was added to react with the sulfhydryl groups on MPTAPs in a one-to-one stoichiometric ratio, producing dissociated molecules (2-nitro-5-thiobenzoic acid, TNB) in the solution and connective disulfides on the solid MPTAPs. After magnetic separation, the TNB solution was introduced into a UV/Vis spectrophotometer for the quantification of TNB molecules, which usually display a specific absorbance peak around 412 nm. As the amount of TNB molecules was equal to the quantity of sulfhydryl groups on MPTAPs, the content of -SH on MPTAPs was measured to be 3.47 mmol g^−1^. Additionally, MPTAPs were characterized by Raman spectrometer and the specific Raman peak of the S-S bond at about 965 cm^−1^ was observed (Figure S1, Supplementary Materials), confirming the formation of connective disulfides, which is realized by the oxidation–reduction reaction between TA’s sodium salt (TMS, TTS) and iodine. The above characterization results of triazine groups, sulfhydryl groups and S-S bonds fully illustrate the successful decoration of TA-based POPs on the Fe_3_O_4_@SiO_2_ magnetic particles.

The magnetism of the prepared materials was assessed by VSM, with the findings illustrated in Figure 2b. The saturation magnetization values for Fe_3_O_4_@SiO_2_ and MPTAPs were determined to be 49.9 and 28.4 emu g^−1^, respectively, which indicates sufficient strength for magnetic separation. The magnetism of MPTAPs is weaker than Fe_3_O_4_@SiO_2_, and this difference is attributed to the increased thickness of the coating on MPTAPs, achieved through the decoration of TA-based POPs upon the surface of Fe_3_O_4_@SiO_2_.

Thermogravimetric analysis of Fe_3_O_4_@SiO_2_ and MPTAPs was conducted to examine the modification result and the content of organic polymer (Figure 2c). Fe_3_O_4_@SiO_2_ exhibited a weight loss of approximately 5%, while MPTAPs showed a much higher weight loss of 64%. The increased weight loss of MPTAPs is attributed to the decomposition of POPs, expected to occur around 260 °C (as indicated by the weight loss observed at approximately 260 °C in Figure 2c). Additionally, the weight loss for MPTAPs remains below 7% until 260 °C, indicating the stability of MPTAPs up to this temperature.

The crystal structures of Fe_3_O_4_@SiO_2_ and MPTAPs were examined using XRD, which revealed six distinct diffraction peaks at 29.95, 35.41, 43.13, 54.04, 57.12 and 63.07 degrees, as shown in Figure 2d. The positions of these peaks are consistent with the reference diffraction pattern for Fe_3_O_4_, which has a cubic spinel structure [28]. No additional XRD peaks were detected in MPTAPs, suggesting the amorphous nature of the external polymer networks.

**Figure 3:**
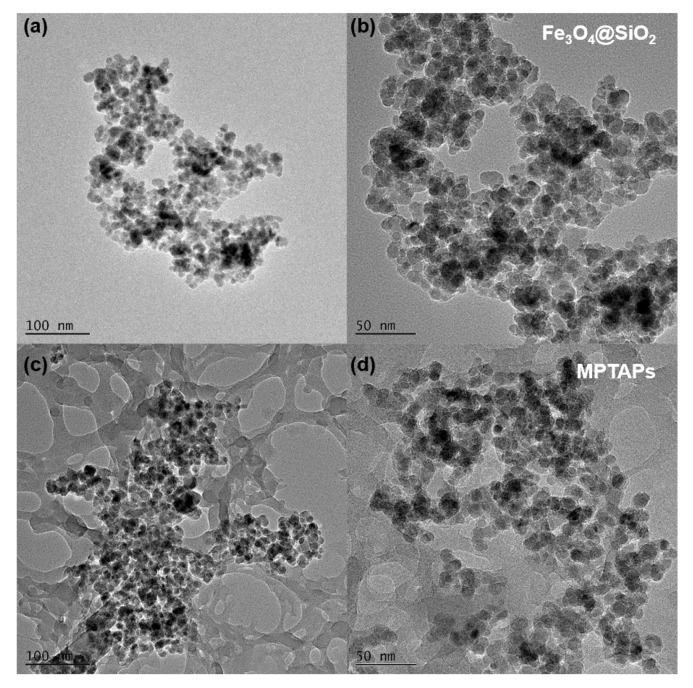
TEM pictures of Fe_3_O_4_@SiO_2_ (**a**,**b**) and MPTAPs (**c**,**d**) (magnifications: (**a**,**c**) 400,000×; (**b**,**d**) 700,000×).

The morphology of Fe_3_O_4_@SiO_2_ and MPTAPs was observed by TEM. As depicted in Figure 3, Fe_3_O_4_@SiO_2_ exhibits a typical core–shell structure, while MPTAPs are composed of inner Fe_3_O_4_@SiO_2_ magnetic nanoparticles and outside large-area porous polymer networks with hierarchical pores (mesopores and macropores). Compared with Fe_3_O_4_@SiO_2_, the different morphology of MPTAPs is attributed to the successful decoration of POPs upon the exterior of Fe_3_O_4_@SiO_2_.

Furthermore, an assessment of the porous property of MPTAPs was conducted through nitrogen adsorption/desorption experiments. The obtained adsorption/desorption isotherm curves of MPTAPs show a characteristic type IV isotherm (Figure S2, Supplementary Materials), and the hysteresis loops are found within the relative pressure range of 0.70~0.95, suggesting the coexistence of mesopores and macropores in MPTAPs. These results align well with the porous networks observed in TEM images of MPTAPs. Furthermore, MPTAPs have a specific pore volume (0.63 cm^3^ g^−1^) and a large BET surface area (124 m^2^ g^−1^). These values significantly surpass those of Fe_3_O_4_@SiO_2_ (0.17 cm^3^ g^−1^ and 35 m^2^ g^−1^), thereby confirming the successful decoration of porous networks on Fe_3_O_4_@SiO_2_. Collectively, these results affirm the successful fabrication of MPTAPs, rendering them suitable for adsorption purposes.

### 3.2. Adsorption Tests

#### 3.2.1. The Study of Adsorption pH and Adsorption Selectivity

**Figure 4:**
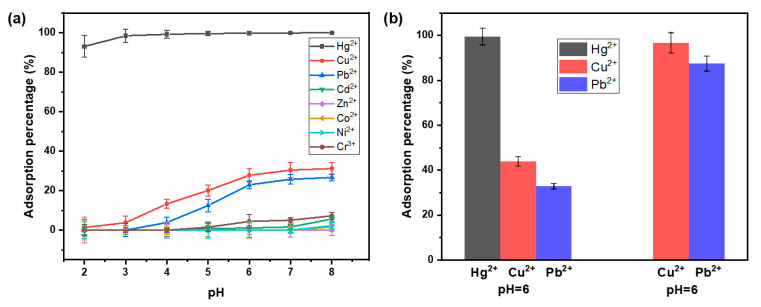
Impact of adsorption pH for MPTAPs toward Hg^2+^ and other co-existing metal ions (**a**) and the adsorption prospect of MPTAPs toward Cu^2+^ and Pb^2+^ (**b**) (concentration of every kind of metal ion: 10 mg L^−1^; solution volume: 50 mL; mass of MPTAPs: 10 mg; adsorption time: 20 min).

Considering that different water bodies may have varying pH values and various coexisting metal ions, we first investigated the influence of solution pH on the adsorption of Hg^2+^ and several common co-existing metal ions (including Pb^2+^, Cd^2+^,Cu^2+^, Zn^2+^, Co^2+^, Ni^2+^ and Cr^3+^) by MPTAPs. As illustrated in Figure 4a, the adsorption percentage of Hg^2+^ on MPTAPs exceeds 90% across the broad pH range of 2~8, while the adsorption rate of MPTAPs for other co-existing metal ions is obviously lower. This result clearly demonstrates the high selective adsorption ability of MPTAPs toward Hg^2+^ in a broad pH range (especially in a highly acidic environment of pH 2~3). This feature ensures the applicability of MPTAPs for the selective separation of Hg^2+^ under various pH environments (acidic or neutral solution). The possible reason for the high selectivity of MPTAPs toward Hg^2+^ may be related to the strong binding ability of the sulfhydryl groups present in MPTAPs toward Hg^2+^, which can be elucidated by Pearson’s theory; soft ligands, such as -SH, readily form complexes with soft metal ions like Hg^2+^ rather than boundary acids (e.g., Pb^2+^) or hard acids (e.g., Co^2+^).

Furthermore, it is important to mention that the adsorption rate of MPTAPs toward Cu^2+^ and Pb^2+^ gradually increased with rising pH, followed by reaching adsorption equilibrium at approximately pH 6, exhibiting a huge adsorption difference in pH dependence against Hg^2+^ on MPTAPs. This distinction is related to their different chelation constants with sulfur [29], as the *K*sp value of HgS remains stable (4 × 10^−53^) across varying pH levels, whereas the *K*sp value of CuS and PbS is highly affected by acidic conditions, and the value of *K*sp_CuS_ (6 × 10^−36^) and *K*sp_PbS_ (1 × 10^−28^) in a neutral environment would reduce to 6 × 10^−15^ and 1 × 10^−6^ under acidic conditions, respectively. The strong adsorption force between Hg^2+^ and sulfhydryl groups in MPTAPs under highly acidic conditions (pH 2~3) makes it possible for MPTAPs to remove Hg^2+^ ions from industrial acidic wastewater, which is highly advantageous for controlling mercury pollution.

To further study the potential adsorption prospect of MPTAPs toward Cu^2+^ and Pb^2+^, the adsorption experiments for MPTAPs toward Cu^2+^ and Pb^2+^ were conducted in the presence and absence of Hg^2+^ ions under pH 6. As depicted in Figure 4b, when Cu^2+^ and Pb^2+^ ions coexist with Hg^2+^ ions, Hg^2+^ ions can be completely adsorbed by MPTAPs, while the adsorption rates of Cu^2+^ and Pb^2+^ are relatively low, verifying the great selectivity of MPTAPs toward Hg^2+^ again. However, in the absence of Hg^2+^ ions, MPTAPs exhibit high adsorption rates of 96.7% for Cu^2+^ and 87.4% for Pb^2+^, suggesting the adsorption ability of MPTAPs for selectively removing Cu^2+^ and Pb^2+^ from the solution without Hg^2+^ ions. Thus, the MPTAP sorbent is also promising for the treatment of Cu^2+^ and Pb^2+^ containing wastewater in a near-neutral environment in the absence of Hg^2+^. The adsorption feasibility test was carried out by mixing 50 mL Cu^2+^ and Pb^2+^ containing industrial wastewater (solution pH was pre-adjusted to 6) with 10 mg MPTAPs. It was found that the adsorption rates of Cu^2+^ and Pb^2+^ were more than 85%, confirming the feasibility of MPTAPs for removing Cu^2+^ and Pb^2+^ from wastewater in the absence of Hg^2+^.

The above adsorption tests confirmed that MPTAPs contain a large number of sulfhydryl groups, indicating that TA-based POPs were successfully modified on the surface of Fe_3_O_4_@SiO_2_. Notably, the great adsorption selectivity of MPTAPs toward Hg^2+^ ions in a broad pH range is beneficial for the selective separation of Hg^2+^ ions under different sample environments. Considering the near-neutral pH value for daily used water and the tendency of metal ions to undergo hydrolysis in alkaline conditions, subsequent adsorption studies were carried out at pH 6.

#### 3.2.2. Adsorption Kinetic Study

**Figure 5:**
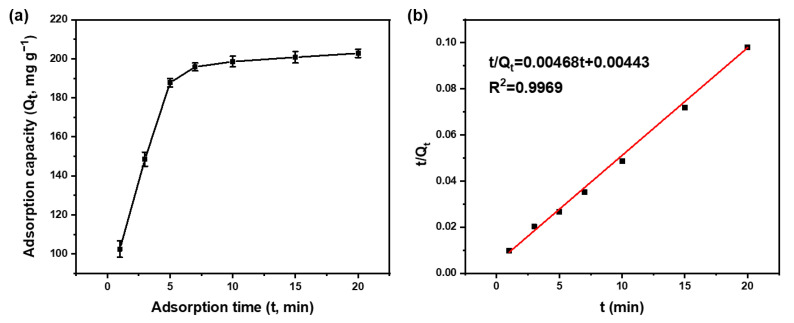
Adsorption kinetics of Hg^2+^ on MPTAPs (**a**) and pseudo-second-order fitted curve (**b**) (mass of MPTAPs: 10 mg; solution pH: 6; concentration of Hg^2+^: 100 mg L^−1^).

To further explore the adsorption ability of MPTAPs toward Hg^2+^, the adsorption kinetic experiments of MPTAPs toward Hg^2+^ were conducted by altering the adsorption time of 1, 3, 5, 7, 10, 15 and 20 min (Figure 5a), while the addition of MPTAPs was fixed as 10 mg and the initial concentration of Hg^2+^ was 100 mg L^−1^. The absorption capacity (Q_t_, mg g^−1^) of Hg^2+^ on MPTAPs at different adsorption times exhibits a rapid increase within the initial 5 min, followed by reaching adsorption equilibrium at approximately 10 min. This result indicates a rapid adsorption process of MPTAPs towards Hg^2+^, which can be attributed to the plentiful high-activity binding sites and hierarchical pores on MPTAPs, offering highly promising prospects for rapidly removing Hg^2+^ ions. For efficient adsorption, the contact time for MPTAPs toward Hg^2+^ was fixed at 10 min in further studies. From the adsorption data in Figure 5a, the value of maximum adsorption capacity of MPTAPs toward Hg^2+^ was measured to be 209 mg g^−1^, which indicate a robust adsorption ability for MPTAPs toward Hg^2+^. To further study the adsorption kinetic process and potential rate-controlling step, the adsorption data were applied to the pseudo-first-order model and pseudo-second-order model, respectively. The pseudo-second-order fitted curve (Figure 5b) shows much better linearity than the pseudo-first-order fitted curve in Figure S3 (Supplementary Materials). Furthermore, the experiment data (Q_e, exp_= 209 mg g^−1^) were roughly equal to the speculative equilibrium value (Q_e, theo_= 214 mg g^−1^) calculated from the pseudo-second-order equation (t/Q_t_ = t/Q_e_ + 1/k_2_Q_e_^2^) and data in Figure 5b. These findings demonstrate that the adsorption of MPTAPs toward Hg^2+^ conforms with the pseudo-second-order model, suggesting that the adsorption is predominantly driven by chemical adsorption with high selectivity.

The adsorption kinetic process for MPTAPs toward Hg^2+^ under acidic conditions (pH 2) was also investigated, and similar results were found. Under acidic conditions, the adsorption equilibrium can be reached in about 10 min, and the maximum adsorption capacity of MPTAPs toward Hg^2+^ was measured to be 187 mg g^−1^. This result verified the stability of chelation constants between Hg^2+^ and sulfur under an acidic environment, which ensures promising prospects for removing Hg^2+^ ions from industrial acidic wastewater. Real industrial acidic wastewater was employed for the verification, and a satisfactory removal rate (92%) was obtained, confirming the feasibility of MPTAPs for scavenging Hg^2+^ from industrial acidic wastewater.

#### 3.2.3. Adsorption Isotherm Study

**Figure 6:**
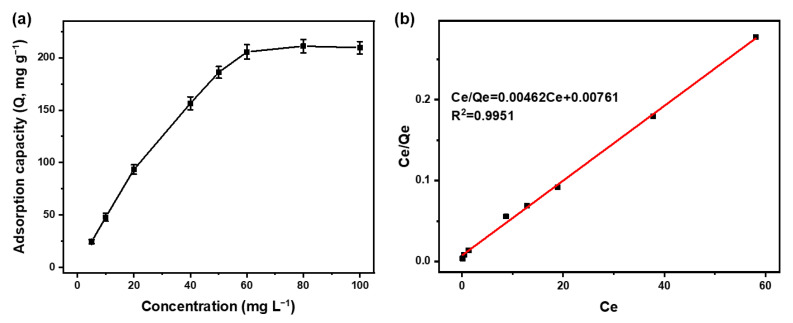
Impact of initial concentration on the adsorption of Hg^2+^ on MPTAPs (**a**) and the fitted Langmuir isotherm (**b**). (mass of MPTAPs: 10 mg; solution pH: 6; adsorption time: 10 min).

To delve deeper into the adsorption mechanism, the impact of initial concentration (5~100 mg L^−1^ Hg^2+^) was examined under adsorption equilibrium conditions with 10 mg MPTAPs mixed with Hg^2+^ solution for 10 min. As shown in Figure 6a, the adsorption capacity of Hg^2+^ rose as the initial concentration increased from 5 to 50 mg L^−1^, after which it leveled off between 60 and 100 mg L^−1^. After calculation, it was measured that the maximum adsorption capacity of Hg^2+^ on MPTAPs was 211 mg g^−1^. Subsequently, the data were applied to fit the equation of the Langmuir model and Freundlich model, respectively. The fitting results showed that the Langmuir isotherm (Figure 6b) had a higher fitting degree with much better linearity than the Freundlich isotherm in Figure S4 (Supplementary Materials). The theoretic maximum adsorption capacity (q_m(theo)_, 216 mg g^−1^) estimated from the Langmuir equation (C_e_/Q_e_ = k_L_/Q_m_ + C_e_/Q_m_) in Figure 6b is near the experimental value (q_m(exp)_, 211 mg g^−1^). These results indicate that the adsorption of MPTAPs toward Hg^2+^ aligns well with the Langmuir model, suggesting a monolayer adsorption mechanism.

### 3.3. Optimization of Extraction Conditions

The impact of the adsorption pH was examined in Section 3.2.1; the quantitative adsorption of Hg^2+^ can be realized under a wide pH range of 2~8. On account of the near-neutral pH value for daily used water and the purpose of reducing hydrolysis behavior of metal ions in an alkaline environment, the following studies were conducted at pH 6. Then, the elution conditions were investigated. In view of the high sulphophile affinity of Hg^2+^ and the lower adsorption rate of Hg^2+^ in acidic solution, the acidic thiourea solution was employed as an eluent for vortex eluting of Hg^2+^ adsorbed by MPTAPs. The curve in Figure S5a (Supplementary Materials) indicates that Hg^2+^ ions can be quantitatively eluted when the thiourea content in 1 mol L^−1^ HCl exceeds 0.2%. To guarantee a higher elution rate, the thiourea content was set as 0.4% in the following studies. Subsequently, the effect of HCl concentration was examined and the curve in Figure S5b (Supplementary Materials) indicates that the recovery rate of Hg^2+^ is above 90% when the concentration of HCl solution exceeds 0.4 mol L^−1^ with 0.4% thiourea. Thus, a 0.5 mol L^−1^ HCl solution containing 0.4% thiourea was used as the optimal eluent in the subsequent experiments for the assurance of quantitative elution. Then, the other elution conditions, including elution volume and elution time, were studied. As can be seen in Figure S5c (Supplementary Materials), Hg^2+^ ions can be quantitatively eluted within the tested elution volume range (0.2~1 mL). In consideration of the purpose of complete elution, 0.4 mL eluent was used in the following studies. The effect of vortex elution time was subsequently examined and the curve in Figure S5d (Supplementary Materials) reveals that the recovery of Hg^2+^ increased rapidly within the first minute and then stabilized after 2 min. To ensure complete elution, the vortex elution period was set to 3 min in subsequent experiments. In short, the optimal elution condition is 0.4 mL of 0.5 mol L^−1^ HCl containing 0.4% thiourea vortex with MPTAPs for 3 min.

Based on the optimized conditions above, the impact of sample solution volume was studied. Several groups of aqueous solution, each containing 10 ng Hg^2+^ and varying in volume from 10 to 100 mL, have been prepared for the adsorption. As depicted in Figure S6a (Supplementary Materials), it is evident that Hg^2+^ ions could be effectively recovered in the tested solution volume range (10~100 mL). To achieve a significant enrichment factor, the sample solution volume was fixed at 100 mL in subsequent experimental procedures. Consequently, the theoretic enrichment factor is calculated to be 250-fold (considering 100 mL sample volume in contrast to the 0.4 mL eluent volume). For the sake of improving the extraction speed, various adsorption times, ranging from 3 to 20 min, were explored under the above conditions. The results indicated that Hg^2+^ could be fully recovered once the extraction time exceeded 5 min, as demonstrated in Figure S6b (Supplementary Materials). To guarantee the complete extraction of trace Hg^2+^ from larger-volume samples, the adsorption time is set as 10 min for the subsequent experiments. Furthermore, the complete recovery of Hg^2+^ could be accomplished by utilizing an adequate quantity of adsorption sorbent. Therefore, the influence of the MPTAP sorbent dosage was examined. As illustrated in Figure S6c (Supplementary Materials), the complete recovery of Hg^2+^ is attainable and was achieved when the sorbent dosage exceeded 5 mg. With the goal of ensuring a comprehensive extraction, the subsequent experiments were conducted using 10 mg of MPTAPs.

### 3.4. Anti-Interference Ability and Recycling Ability

A variety of frequently encountered ions (like K^+^, Na^+^, Fe^3+^, Al^3+^ and Cl^−^ etc.), each present at different concentrations, were introduced into the solution containing 10 ng of Hg^2+^. Subsequently, the extraction process was carried out to assess the impact of these common coexisting ions on the extraction of the target Hg^2+^, using the previously optimized MSPE conditions. The highest permissible concentrations for these coexisting ions are detailed in Table S2 (Supplementary Materials). It was noted that the presence of a high concentration of these coexisting ions had minimal to no effect on the extraction of the target Hg^2+^. Moreover, the tolerance concentrations were found to be several thousand times greater than those of the target Hg^2+^ ions, which underscores the robust interference resistance of the developed MSPE-ICP-MS method.

To assess the reuse potential of MPTAPs, a regeneration process was conducted after the elution step, which involved cleaning the adsorbent with ultrapure water followed by ammonium acetate aqueous solution (10 mmol L^−1^). This sequence of adsorption, elution, and regeneration was repeated multiple times. As depicted in the trends displayed in Figure S7 (Supplementary Materials), MPTAPs exhibit the ability to be recycled 10 times without an obvious reduction in recovery. The anti-interference characteristic and reusability reveal that MPTAP is a recyclable adsorbent with high selectivity and efficiency. Thus, MPTAPs were employed as a MSPE adsorbent for effective separation and preconcentration of trace Hg^2+^ from aqueous solution, followed by ICP-MS measurement.

### 3.5. Comparison of Other Sulfur-Containing Adsorbents with MPTAPs

A comparison of other sulfur-containing adsorbents and the prepared MPTAPs is provided in Table 1. The adsorption kinetics (10 min) of MPTAPs toward Hg^2+^ is faster than those adsorbents (15~20 min) reported in Refs. [6,30] and is comparable with other adsorbents in Refs. [31,32]. The adsorption capacity for MPTAPs toward Hg^2+^ (211 mg g^−1^) is much higher than that of other adsorbents in Refs. [31,33], which is attributed to the large surface area and high S content of MPTAPs. Compared with the adsorbents reported in Ref [6,32], MPTAPs have a better reuse performance, up to 10 times. Furthermore, in the preparation step of the disulfide polymer for MPTAPs, no additional harmful organic solvent is required. However, the other reported sulfur-containing adsorbents [6,30,31,32,33] inevitably need to use organic solvents or multi-step synthetic steps in the modification of S-containing monomer. Therefore, it is encouraging that MPTAPs prepared by the green and mild method have a satisfactory adsorption performance, which renders it a promising adsorbent for selective separation of Hg^2+^ ions.

### 3.6. Analytical Performance of the Developed MSPE-ICP-MS Method

The analytical performance of the developed MSPE-ICP-MS method was assessed under the optimized conditions, with the findings presented in Table 2. A strong linear correlation (R^2^ = 0.9981) for Hg^2+^ was observed across the concentration range from 2 to 3000 ng L^−1^. The actual preconcentration factor (PF) for Hg^2+^, which is 232, was calculated by comparing the slopes of the standard curves derived with and without the preconcentration process. The limit of detection (LOD) for Hg^2+^ was determined to be 0.61 ng L^−1^. Comparing the HJ 597-2011 standard method of <<Water quality-Determination of Total mercury-Cold atomic absorption spectrophotometry>> with the LOD of 0.06 μg L^−1^ [34], our proposed MSPE-ICP-MS method exhibited a much lower detection limit, indicating a high sensitivity of the proposed MSPE-ICP-MS method. Furthermore, the intra-assay relative standard deviation (RSD, n = 7, C_Hg_=10 ng L^−1^) and the inter-assay RSD (n = 5, C_Hg_ = 10 ng L^−1^) were 5.7% and 8.5%, respectively, revealing the good reproducibility of our method.

The evaluation of the MPTAP-based MSPE-ICP-MS method is contrasted with various other reported analytical approaches in Table 3. The LOD for our method is on par with those found in the literatures, cited in Refs. [31,32,33]. Moreover, it is lower than the values reported in Refs. [30,35,36,37,38,39], which underscores the method’s commendable sensitivity. Compared with the methodologies reported in Refs. [30,35,37,38,39], the method we have devised is straightforward to execute, and the duration required for sample preparation, a mere 13 min, is significantly reduced. Furthermore, the adsorption capacity of MPTAPs toward Hg^2+^ was higher than that of Fe_3_O_4_@SiO_2_@γ-MPTMS [31], Fe_3_O_4_@SiO_2_@GMA-S-SH [33], Apt-Fe_3_O_4_-SiO_2_-NH_2_@HKUST-1 [37] and biosorbent-modified XAD-4 resin [39], indicating the great potential for MPTAPs in removing toxic Hg^2+^ ions. Overall, the proposed MSPE-ICP-MS analytical technique, which leverages the MPTAP sorbent, is characterized by speed, and high selectivity and sensitivity toward Hg^2+^. It is also eco-friendly and facilitates straightforward phase separation, owing to the magnetic property and recycling ability of MPTAPs. These merits render it appropriate for the analysis of trace Hg^2+^ in aqueous samples.

### 3.7. Real Sample Analysis

An external calibration method was employed to quantify trace Hg^2+^ in aqueous samples, with the analytical outcomes are shown in Table 4. None of the Hg^2+^ was detected in tap water, while Hg^2+^ ions at the sub-µg L^−1^ level were detected in laboratory wastewater, lake water and river water. Spiking experiments were performed to verify the efficacy of the proposed method. The recoveries of Hg^2+^ obtained from the spiked samples, which were satisfactory and within the range of 88.2% to 99.3%, confirmed the method’s suitability for accurately determining trace levels of Hg^2+^ in real water samples using the MSPE-ICP-MS technique. The precision of this method was also confirmed by analyzing Hg^2+^ in the certified reference material of GSB 07-3173-2014 (202061) standard water samples (pre-diluted 10-fold). The results, as presented in Table S3 (Supplementary Materials), were in close alignment with the certified values, indicating the method’s high degree of accuracy.

## 4. Conclusions

In this work, a new MSPE adsorbent, MPTAP, was fabricated through a simple oxidation–reduction reaction between TA’s sodium salt and iodine under aqueous solution with the presence of magnetic nanoparticles. This approach is cost-effective, green, environmentally friendly, gentle and dispenses with harmful solvents and expensive reagents. The resultant MPTAPs feature a highly porous structure and large surface area with abundant sulfhydryl groups, and they exhibit great adsorption affinity toward Hg^2+^ with high selectivity, rapid adsorption kinetics (10 min), large adsorption capacity (211 mg g^−1^) and wide adsorption applicability under various pH environments. Even in an acidic environment (pH 2), MPTAPs still maintain efficient adsorption performance for Hg^2+^ and can be applied for the removal of Hg^2+^ in acidic wastewater. More importantly, MPTAPs can be recycled up to 10 times, which is beneficial for the economy of wastewater treatment or trace elemental analysis. The fast and convenient magnetic separation step for MPTAPs can save much more energy compared with the centrifugal procedure and filtration steps for non-magnetic adsorbents. Hence, the prepared MPTAPs show promising prospects in the rapid and selective separation of Hg^2+^, and a method of MPTAPs-based MSPE technique combined with ICP-MS measurement was constructed for the detection of trace Hg^2+^ in aqueous samples. Under optimized conditions, the MSPE-ICP-MS method based on MPTAPs is efficient, rapid, sensitive and selective for the determination of trace Hg^2+^, which was successfully employed for the accurate analysis of trace Hg^2+^ in tap water, wastewater, lake water and river water samples. Additionally, MPTAPs sorbent is also efficient for the removal of Cu^2+^ and Pb^2+^ from near-neutral wastewater in the absence of Hg^2+^, revealing the great adsorption ability of MPTAPs under various environments.

## Figures and Tables

**Table 1:** Comparison of sulfur-containing adsorbents for adsorption of Hg^2+^ ions.

Adsorbents	Adsorption Capacity (mg g^−1^)	Adsorption Kinetics (min)	Surface Area (m^2^ g^−1^)	S Content (mmol g^−1^)	Times Reused	Organic Solvent ^a^	Ref.
SH-SiO_2_/Cu_3_(BTC)_2_	210	15	/	3.9	15	+	[30]
Fe_3_O_4_@SiO_2_@γ-MPTMS	84	10	/	/	10	+	[31]
Thiol-graftedmagnetic polymer	254	10	156.8	1.7	3	+	[32]
Fe_3_O_4_@COF_TAPB-DEBD_@SH	-	20	136.6	0.24	6	+	[6]
Fe_3_O_4_@SiO_2_@GMA-S-SH	141	5	79.8	0.8	30	+	[33]
MPTAPs	211	10	124	3.47	10	−	This work

Notes: a: +/−, with or without consumption of organic solvent in the modification of S-containing monomer.

**Table 2:** Analytical performance of the proposed MSPE-ICP-MS method.

Target Ion	Regression Equation	R^2^	Concentration Range (ng L^−1^)	PF	LOD (ng L^−1^)
Hg^2+^	*y* = 2436*x* + 6399	0.9981	2~3000	232	0.61

**Table 3:** Comparison of different methods in the adsorption and analysis of Hg^2+.^

Methods	Sorbents	Adsorption Capacity (mg g^−1^)	LOD (ng L^−1^)	PF	Pretreatment Time (min)	Ref.
SPE-CVAAS	SH-SiO_2_/Cu_3_(BTC)_2_	210	20,000	167	>35	[30]
dSPE-ICP-OES	Pectin-coated magnetic graphene oxide	-	1200	50	30	[35]
MSPE-ICP-MS	Fe_3_O_4_@SiO_2_@γ-MPTMS	84	0.1	400	13	[31]
MSPE-ICP-MS	Thiol-grafted magnetic polymer	254	0.82	150	15	[32]
MSPE-ICP-MS	Fe_3_O_4_@COF_TAPB-DEBD_@SH	-	0.51	100	22	[6]
MSPE-HPLC- ICP-MS	Fe_3_O_4_@SiO_2_@GMA -S-SH	141	1.6	400	7	[33]
DLLME-GFAAS	-	-	4.3	100	11	[36]
MSPE-DLLME- GFAAS	Apt-Fe_3_O_4_- SiO_2_- NH_2_@HKUST-1	156	340	2400	37	[37]
SPE-ICP-OES	*Agaricus augustus* Immobilized Fe_2_O_3_	47.2	16	100	168	[38]
SPE-ICP-OES	Biosorbent-modified XAD-4 resin	27.8	60	80	202.5	[39]
MSPE-ICP-MS	MPTAPs	211	0.61	232	13	This work

**Table 4:** Determination results of Hg^2+^ in aqueous samples.

Sample	Added (ng L^−1^)	Found (ng L^−1^)	Recovery (%)	Sample	Added (ng L^−1^)	Found (ng L^−1^)	Recovery (%)
Tap water	0	/	/	River water	0	9.3 ± 1.2	/
	50	48.7 ± 3.1	97.4		50	57.4 ± 3.7	96.2
	200	189 ± 8	94.5		200	201 ± 9	95.9
	1000	993 ± 12	99.3		1000	987 ± 16	97.8
Waste water	0	181 ± 14	/	Lake water	0	2.6 ± 0.5	/
	50	228 ± 19	94.0		50	48.9 ± 4.2	92.6
	200	374 ± 23	96.5		200	179 ± 9	88.2
	1000	1132 ± 47	95.1		1000	960 ± 21	95.7

## Data Availability

Data are contained within the article and Supplementary Materials.

## Supplementary Materials

The following supporting information can be downloaded at: https://www.mdpi.com/article/10.3390/polym16213067/s1, Figure S1: Raman spectra characterization of MPTAPs; Figure S2: Nitrogen adsorption/desorption isotherm and pore distribution of MPTAPs; Figure S3: The pseudo-first-order kinetic plot for the adsorption of Hg^2+^ on MPTAPs; Figure S4: The Freundlich isotherm for the adsorption of Hg^2+^ on MPTAPs; Figure S5: Effect of thiourea content (a), HCl concentration (b), elution volume (c) and elution time (d) on the recovery of Hg^2+^ extracted by MPTAPs; Figure S6: Effect of sample volume (a), adsorption time (b) and mass of material (c) on the recovery of Hg^2+^ on MPTAPs; Figure S7: Effect of reuse times on the recovery of Hg^2+^ on MPTAPs; Table S1: Working conditions for ICP-MS analysis; Table S2: Tolerance limits of coexisting ions; Table S3: Analytical results of Hg in certified reference material; Appendix SA: Preparation of MNPs of Fe_3_O_4_@SiO_2_. References [31,40,41] are cited in the supplementary materials.
