# Supplementary material for: Green and Mild Fabrication of Magnetic Poly(trithiocyanuric acid) Polymers for Rapid and Selective Separation of Mercury(II) Ions in Aqueous Samples
Source: Polymers (Basel). 2024 Oct 31;16(21):3067. doi: 10.3390/polym16213067 (PMC11548154; doi:10.3390/polym16213067)
Supplement: Supplementary file 1 [file polymers-16-03067-s001.zip › polymers-3270756-supplementary.pdf]

polymers

MDPI

Supplementary Materials

Green and Mild Fabrication of Magnetic Poly(trithiocyanuric acid) Polymers for Rapid and Selective Separation of Mercury(II) Ions in Aqueous Samples

Qianqian Li, Boxian Ruan, Yue Yu, Linshu Ye, Aoxiong Dai, Sasha You, Bingshan Zhao * and Limin Ren *

Hubei Key Laboratory of Processing and Application of Catalytic Materials, Department of Chemistry, Huanggang Normal University, Huangzhou 438000, China; liqianqian@hgnu.edu.cn (Q.L.)
* Correspondence: zhaobingshan@hgnu.edu.cn (B.Z.); fulilimin@163.com (L.R.)

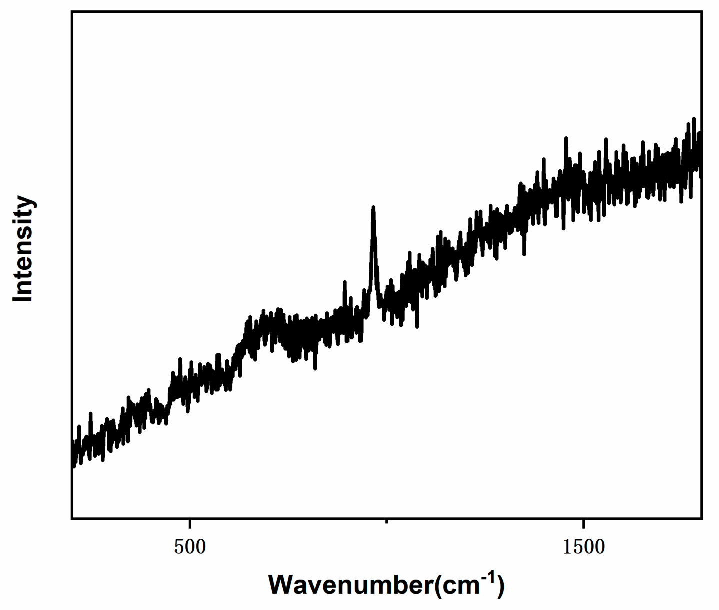

Figure S1. Raman spectra characterization of MPTAPs.

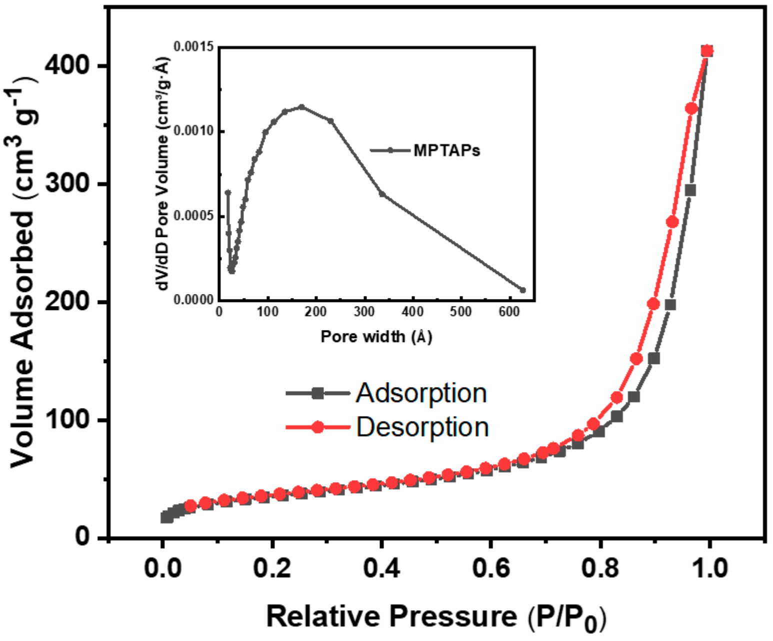

Figure S2. Nitrogen adsorption/desorption isotherm and pore distribution of MPTAPs.

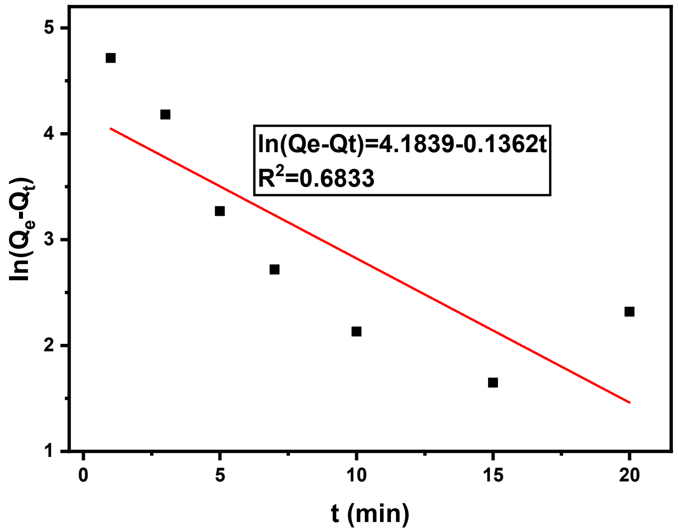

Figure S3. The pseudo-first-order kinetic plot for the adsorption of Hg^{2+} on MPTAPs.

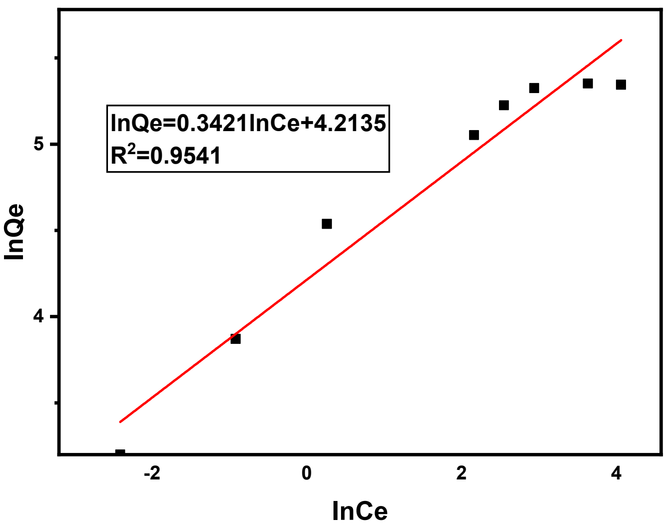

Figure S4. The Freundlich isotherm for the adsorption of Hg^{2+} on MPTAPs.

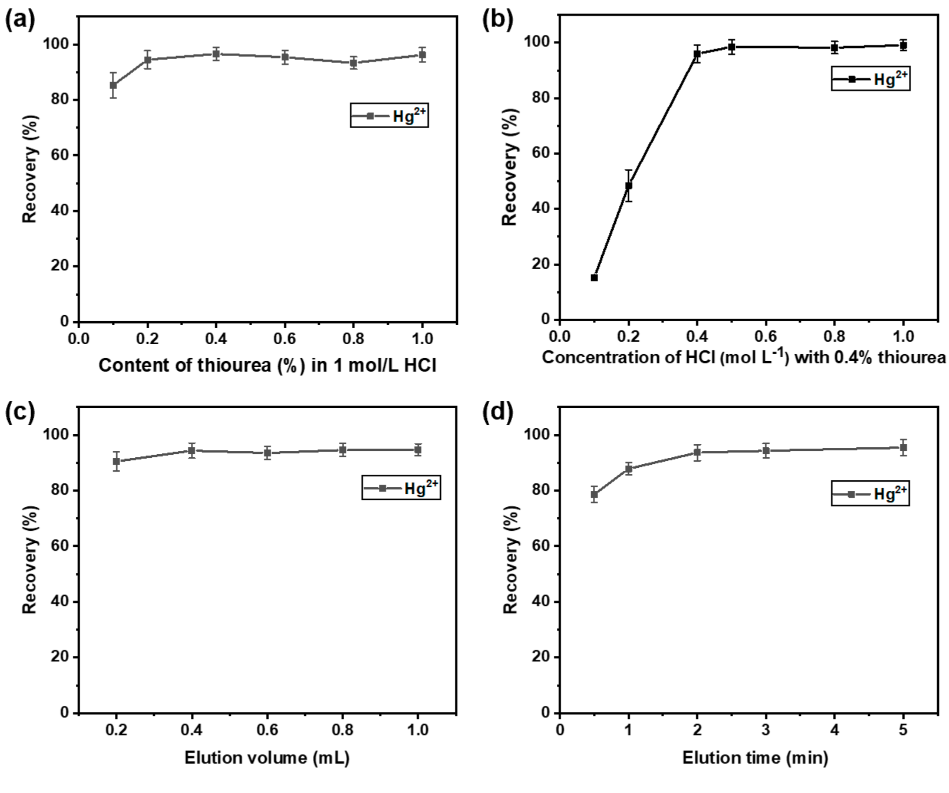

Figure S5. Effect of thiourea content (a), HCl concentration (b), elution volume (c) and elution time (d) on the recovery of Hg^{2+} extracted by MPTAPs. Conditions: pH, 6; mass of Hg^{2+}, 10 ng; adsorption time, 20 min; elution conditions, 0.5 mL 1 mol L^{-1} HCl + thiourea eluted for 20 min (a), 0.5 mL HCl+0.4% thiourea eluted for 20 min (b), 0.2~1 mL 0.5 mol L^{-1} HCl+0.4% thiourea eluted for 20 min (c); 0.4 mL 0.5 mol L^{-1} HCl+0.4% thiourea eluted for 0.5~5 min (d). Error bars show the standard deviation (n = 3).

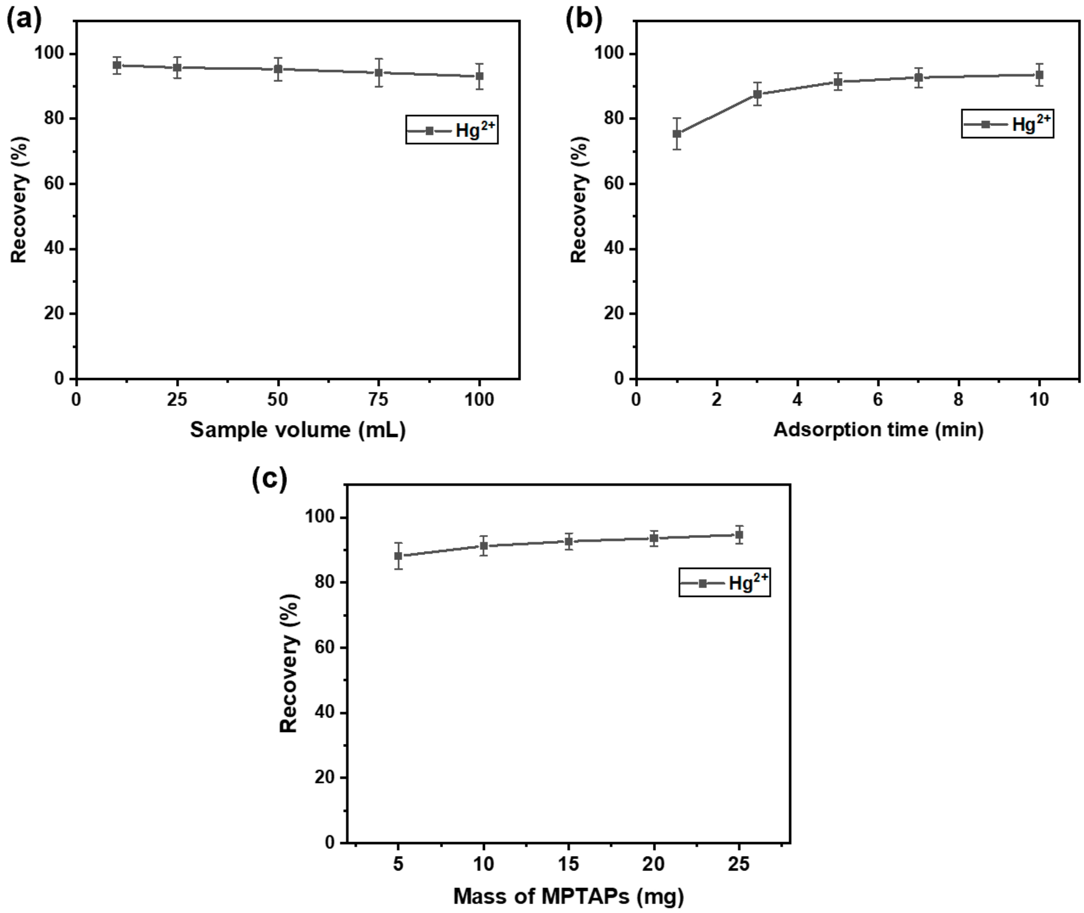

Figure S6. Effect of sample volume (a), adsorption time (b) and mass of material (c) on the recovery of Hg^{2+} on MPTAPs. Conditions: sorbents, 5 mg; pH, 6; content of Hg^{2+}, 10 ng; sample volume, 10~100 mL (a),100 mL (b,c); extraction time, 20 min (a,b), 5~20 min (c); eluent, 0.5 mol L^{-1} HCl+0.4% thiourea; elution volume, 0.4 mL; elution time, 3 min. Error bars show the standard deviation (n = 3).

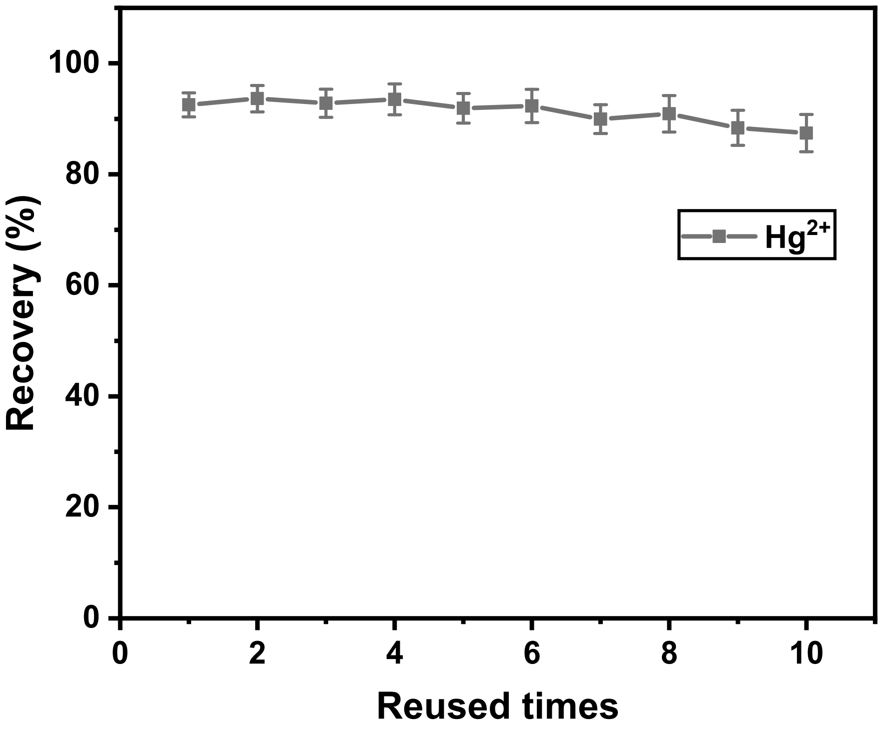

Figure S7. Effect of reuse times on the recovery of Hg^{2+} on MPTAPs. Conditions: materials, 10 mg; pH, 6; mass of Hg^{2+}, 10 ng; sample volume, 100 mL; adsorption time, 10 min; eluent, 0.5 mol L^{-1} HCl+0.4% thiourea; elution volume, 0.4 mL; elution time, 3 min. Error bars show the standard deviation (n = 3).

Table S1. Working conditions for ICP-MS.

ICP-MS parameters	
Power	1200 W
Atomizer	Glass concentric atomizer
Torch tube	Mini torch tube system
Peristaltic pump speed	20 r.p.m
Sampling depth	5 mm
Plasma gas	8.0 L min^{-1}
Auxiliary gas	1.1 L min^{-1}
Carrier gas flow	0.7 L min^{-1}
He gas flow	6 mL/min
Number of repeated measurements	3
Integration time	2 s

Table S2. Tolerance limits of coexisting ions (C_{Hg}=10 ng L^{-1}).

Coexisting Ions	Tolerance Limits of Coexisting Ions (mg L^{-1})	Average Concentration of Common Ions in Natural Water (mg L^{-1}) [1]
K^+	5000	1.6
Na^+	5000	11
Ca^{2+}	2500	95
Mg^{2+}	2500	38
Fe^{3+}	10	--
Al^{3+}	10	--
Cu^{2+}	20	--
Zn^{2+}	20	--
Cl^-	7500	25
NO_3^-	8000	--
SO_4^{2-}	12,000	71.5

Table S3. Analytical results of Hg in certified reference material.

Element	Certified Reference Material	Certified Value	Determined Value [a]	t Test [b]
Hg	GSB 07-3173-2014 (μg L^{-1})	7.5 ± 0.7	7.3 ± 0.4	0.87

Notes: [a], the determined value were presented as mean value ± standard deviation (n = 3). [b], $t_{0.05,2}$ = 4.31.

Appendix SA: Preparation of $Fe_3O_4@SiO_2$ MNPs

First, Fe_3O_4 nanoparticles were synthesized by coprecipitation method [2] with minor modifications. Briefly, 11.68 g $FeCl_3{\cdot}6H_2O$ and 4.30 g $FeCl_2{\cdot}4H_2O$ were dissolved in 200 mL high purity deionized water and heated to 85 °C under nitrogen gas. After heating at 85 °C for 1 h, 25 mL $NH_3{\cdot}H_2O$ (28%, v/v) was added with vigorous stirring for 0.5 h. The obtained Fe_3O_4 nanoparticles were washed several times with high purity deionized water and ethanol, and stored in 200 mL ethanol at a concentration of about 20 mg mL^{-1}. Subsequently, $Fe_3O_4@SiO_2$ were prepared as a similar way in Ref. [3]. 100 mL magnetic fluid of Fe_3O_4 prepared above was homogeneously dispersed in a mixture of ethanol (100 mL) and high purity deionized water (20 mL) by sonication for 15 min, followed by the addition of 3 mL $NH_3{\cdot}H_2O$ (28%, v/v) and 3 mL TEOS sequentially. After being stirred at room temperature for 12 h, the resultant $Fe_3O_4@SiO_2$ were rinsed with high purity deionized water and ethanol for several times, then dried in vacuum at 60 °C overnight.

References

1. Zhang, L.; Chen, B.; He, M.; Liu, X.; Hu, B. Hydrophilic polymer monolithic capillary microextraction online coupled to ICPMS for the determination of carboxyl group-containing gold nanoparticles in environmental waters. *Anal. Chem.* **2015**, *87*, 1789–1796.
2. Huang, C.; Hu, B. Silica-coated magnetic nanoparticles modified with γ-mercaptopropyltrimethoxysilane for fast and selective solid phase extraction of trace amounts of Cd, Cu, Hg, and Pb in environmental and biological samples prior to their determination by inductively coupled plasma mass spectrometry. *Spectrochim. Acta Part B* **2008**, *63*, 437–444.
3. Zhao, B.; He, M.; Chen, B.; Xu, H.; Hu, B. Poly(1-vinylimidazole) functionalized magnetic ion imprinted polymer for fast and selective extraction of trace gold in geological, environmental and biological samples followed by graphite furnace atomic absorption spectrometry detection. *Spectrochim. Acta Part B At. Spectrosc.* **2018**, *143*, 32–41.
